# High expression of miR-363 predicts poor prognosis and guides treatment selection in acute myeloid leukemia

**DOI:** 10.1186/s12967-019-1858-7

**Published:** 2019-04-01

**Authors:** Huihui Zhang, Ninghan Zhang, Rong Wang, Tingting Shao, Yuan Feng, Yao Yao, Qingyun Wu, Shengyun Zhu, Jiang Cao, Huanxin Zhang, Zhenyu Li, Xuejiao Liu, Mingshan Niu, Kailin Xu

**Affiliations:** 1 Blood Diseases Institute, Xuzhou Medical University, Xuzhou, Jiangsu China; 2 Department of Hematology, Affiliated Hospital of Xuzhou Medical University, Xuzhou, Jiangsu China; 3 Jiangsu Key Laboratory of Bone Marrow Stem Cell, Xuzhou Medical University, Xuzhou, Jiangsu China; 4 Insititute of Nervous System Diseases, Xuzhou Medical University, Xuzhou, Jiangsu China

**Keywords:** Mir-363, Acute myeloid leukemia, Clinical outcome, Chemotherapy, Allo-HSCT

## Abstract

**Background:**

Acute myeloid leukemia (AML) is a highly heterogeneous malignancy with various outcomes, and therefore needs better risk stratification tools to help select optimal therapeutic options.

**Methods:**

In this study, we identify miRNAs that could predict clinical outcome in a heterogeneous AML population using TCGA dataset.

**Results:**

We found that MiR-363 is a novel prognostic factor in AML patients undergoing chemotherapy. In multivariable analyses, high miR-363 remained predictive for shorter OS (HR = 2.349, P = 0.012) and EFS (HR = 2.082, P = 0.001) independent of other well-known prognostic factors. More importantly, allogeneic hematopoietic stem cell transplantation (allo-HSCT) overcame the adverse outcomes related to high miR-363 expression. In gene expression profiling, high miR-363 expression was positively correlated with the amounts of leukemogenic transcription factors, including Myb, RUNX3, GATA3, IKZF3, ETS1 and MLLT3. Notably, we found that the in silico predicted target genes (EZH2, KLF6 and PTEN) of miR-363 were downregulated in association with high miR-363 expression.

**Conclusions:**

In summary, miR-363 expression may help identify patients in need of strategies to select the optimal therapy between chemotherapeutic and allo-HCST regimens. AML patients with high miR-363 expression may be highly recommended for early allo-HSCT regimen.

## Background

Acute myeloid leukemia (AML), the most frequent form of acute leukemia in adults, is caused by a rapid clonal proliferation of neoplastic myeloblasts [1]. Patients with AML manifest complex and heterogeneous outcomes after receiving different treatments. Conventional cytotoxic treatment with chemotherapeutics is the first-line therapy in AML [2]. High-risk patients could receive effectively antileukemic action and potential cure after accepting allogeneic hematopoietic stem cell transplantation (allo-HSCT). The variation in AML patient prognosis is related to various inherent factors, including cytogenetics and genetic alterations. Somatic mutations in NPM1, CEBPA, FLT3, IDH1, IDH2 and TET2 are associated with outcomes of patients and served as prognostic markers in AML [3]. Despite the molecular mechanisms of leukemogenesis are well known, most AML patients are not cured. Notably, the currently available risk stratification systems are not completely accurate. Therefore, novel prognostic markers are needed to improve AML risk classification and select optimal therapeutic schedule.

MicroRNAs (miRNAs) represent short noncoding RNAs, which hybridize to target mRNAs with high specificity and decrease protein levels through translation inhibition [4]. Dysregulation of miRNAs expression in AML can affect cell proliferation, survival and hematopoietic differentiation [5]. More importantly, abnormal expression of miRNAs is related to clinical outcomes of AML patients. For instance, high miR-181a level has been confirmed to predict favorable survival in cytogenetically normal AML cases [6]. Patients with high miR-212 level tend to have better outcome independently of cytogenetic subtype [7]. Moreover, high miR-3151 expression is associated with worse overall survival and disease-free survival in patients cytogenetically normal AML [8]. However, the majority of previous studies did not distinguish the various effects of chemotherapy and allo-HSCT treatment on the therapeutic outcome. As is well known, the prognostic impact of a marker is treatment-dependent in AML. Consequently, miRNAs may have different prognostic effects in chemotherapy and allo-HSCT treatment, respectively.

In this study, we identify miR-363 that could predict clinical outcome in a heterogeneous AML population using genome-wide screening. The prognostic role of miR-363 is independent of known potent clinical and molecular predictors. The miR-363 expression contributed to risk classification in AML patients undergoing chemotherapy. We also evaluated whether allo-HCST could overcome the poor prognostic effects of high miR-363 level in the same cohort. In order to evaluate biological insights of miR-363, we performed genome-wide gene and miRNA expression analyses.

## Materials and methods

### Patients

We studied a total of 162 patients with newly diagnosed AML according to the WHO classification. The RNA-Seq expression data of these AML patients were provided by The Cancer Genome Atlas (TCGA) [9]. This study has been approved by Human Studies Committee of the Washington University. Patients with AML were included in a single center’s tissue protocol and followed NCCN guidelines to receive treatment. Patients with unfavorable risk underwent allo-HSCT if they were medically fit for the risks of transplantation, and if a suitably matched donor was available. In this cohort, 90 patients were only treated by chemotherapy and another 72 patients accepted both chemotherapy and allo-HSCT. All clinical data are available on the TCGA website.

### Gene-expression profiling

Of the 162 patients, only 155 had both microRNA and mRNA expression data. For mRNA-seq data, genes expressed at or below a noise threshold of RPKM (Reads per kilobase per million mapped reads) ≤ 0.2 in at least 75% of samples were removed. For miRNA-seq data, read counts were normalized to RPM (Reads per million reads). The expression data were log2 transformed before analysis. The gene/microRNA expression signatures were derived by Spearman correlation analysis (Benjamini–Hochberg adjusted *P* value < 0.01). Finally, gene rows were reordered using hierarchical clustering analysis. The miRBase Targets Version 7 and Targetscan Release 7.1 were employed to predict the targets of miR-363. Gene Ontology enrichment assessment of genes in miR-363 related signature was conducted with the Database for Annotation, Visualization, and Integrated Discovery (DAVID).

### Statistical analysis

A comparison of baseline characteristics between patients with high and low miRNA expression patients was conducted. The median miR-363 level was used to identify patients with low and high miRNA expression, respectively. Mann–Whitney U test was performed to test relations between two continuous variables. Fisher’s exact and Chi square tests were determined for categorical variables. Overall survival (OS) was the time from patient diagnosis to death at the final follow-up. Event-free survival (EFS) was the time from patient diagnosis to adverse events, including relapse and death. Kaplan–Meier method was performed to evaluate OS and EFS distributions and the log-rank test was employed to compare survival curves.

Univariable Cox proportional hazards models were constructed for assessing correlations of miR-363 expressions with OS and EFS, respectively. We establish multivariable Cox proportional hazards models to identify factors affecting OS and EFS. The factors included in the evaluation model contained miR-363 expression levels, FLT3–ITD, NPM1, DNMT3A, RUNX1, TP53, TET2, MLL–PTD, IDH1/IDH2 and NRAS/KRAS mutation statuses, and WBC involvement. Factors showing significance with α = 0.20 in univariable analysis were entered into limited backward selection to generate multivariable models. Variables remaining in the final models were significant at α = 0.05. The R software 3.1.5, GraphPad Prism and SPSS were used for statistical analysis, with *P* < 0.05 indicating statistical significance.

## Results

### Association of miR-363 level with clinico-molecular properties

The patients were divided into two groups, chemotherapy and allo-HSCT groups. Subsequently, each group was subdivided into two groups in accordance with the median of miR-363. The relationship between clinical-genetic characteristics and miR-363 expression is shown in Table 1. In patients who underwent chemotherapy, cases with high miR-363 levels showed higher relapse rate (*P* = 0.001), and lower WBC count (*P* = 0.001) and circulating blast amounts (*P* = 0.007) at initial diagnosis in comparison with those expressing low miR-363 amounts. Patients with elevated miR-363 expression comprised less cases with favorable risk (*P* = 0.002), but more with poor risk of AML (*P* = 0.018). Furthermore, patients with high miR-363 expression included 92% of all cases with complex karyotypes and all cases with TP53 gene mutation. Meanwhile, Low miR-363 expressers encompassed all cases with the CBFβ-MYH11 fusion gene and 69% of all cases with NPM1 gene mutation.

**Table 1:** Comparison of clinical and molecular characteristics with miR-363 expression in AML patients

Characteristic	Chemotherapy group			Allo-HSCT group		
	High miR-363 (n = 45)	Low miR-363 (n = 45)	*P*	High miR-363 (n = 36)	Low miR-363 (n = 36)	*P*
Age/years, median	68 (33–88)	62 (22–77)	0.005	52 (18–65)	51 (21–72)	0.585
Age group, n (%), years			0.006			0.793
< 60	8 (17.8)	21 (46.7)		25 (69.4)	27 (75.0)	
≥ 60	37 (82.2)	24 (53.3)		11 (30.6)	9 (25.0)	
Gender, n (%)			1.000			0.634
Male	25 (55.6)	25 (55.6)		22 (55.6)	19 (52.8)	
Female	20 (44.4)	20 (44.4)		14 (38.9)	17 (47.2)	
WBC, × 10^9^/L, median	8.3 (0.7–171.9)	39.8 (2.5–298.4)	0.001	11.4 (0.6–77.3)	35.8 (1.2–223.8)	0.001
BM blast (%), median	71 (30–98)	73 (32–99)	0.580	67.5 (30–95)	71.5 (39–100)	0.156
PB blast (%), median	16 (0–91)	52 (0–98)	0.007	33.5 (0–90)	56 (8–96)	0.008
FAB subtypes, n (%)						
M0	5 (11.1)	3 (6.7)	0.714	7 (19.4)	2 (5.6)	0.151
M1	9 (20)	11 (24.4)	0.800	10 (37.8)	13 (36.1)	0.614
M2	10 (22.2)	11 (24.4)	1.000	9 (25.0)	10 (27.8)	1.000
M4	10 (22.2)	14 (31.1)	0.475	5 (13.9)	9 (25.0)	0.372
M5	8 (17.8)	5 (11.1)	0.550	3 (8.3)	1 (2.8)	0.614
M6	2 (4.4)	1 (2.2)	1.000	1 (2.8)	0	1.000
M7	1 (2.2)	0 (0)	1.000	1 (2.8)	0	1.000
Others	1 (2.2)	0 (0)	1.000	0	1 (2.8)	1.000
Karyotype, n (%)						
Normal	20 (44.4)	24 (53.3)	0.527	16 (44.4)	18 (50.0)	0.814
Complex	11 (24.4)	1 (2.2)	0.004	11 (30.6)	1 (2.8)	0.003
8 Trisomy	0	2 (4.4)	0.494	2 (5.6)	5 (13.9)	0.429
CBFβ–MYH11	0	7 (15.6)	0.012	0	5 (13.9)	0.054
11q23/MLL	4 (8.9)	1 (2.2)	0.361	2 (5.6)	1 (2.8)	1.000
−7/7q−	2 (4.4)	1 (2.2)	1.000	1 (2.8)	1 (2.8)	1.000
BCR–ABL1	1 (2.2)	0	1.000	1 (2.8)	1 (2.8)	1.000
RUNX1–RUNX1T	1 (2.2)	5 (11.1)	0.203	0	1 (2.8)	1.000
Others	6 (13.3)	4 (8.9)	0.739	3 (8.3)	3 (8.3)	1.000
Risk, n (%)						
Good	1 (2.2)	12 (26.7)	0.002	0	6 (16.7)	0.025
Intermediate	25 (55.6)	25 (55.6)	1.000	19 (52.8)	22 (61.1)	0.634
Poor	18 (40.0)	7 (15.6)	0.018	17 (47.2)	7 (19.4)	0.023
Others	1 (2.2)	1 (2.2)	1.000	0	1 (2.8)	1.000
FLT3–ITD, n (%)			0.784			0.045
Presence	7 (15.6)	9 (20.0)		4 (11.1)	12 (33.3)	
Absence	38 (84.4)	36 (80.0)		32 (88.9)	24 (66.7)	
NPM1, n (%)			0.023			0.430
Mutation	9 (20.0)	20 (44.4)		8 (22.2)	12 (33.3)	
Wild type	36 (80.0)	25 (55.6)		28 (77.8)	24 (66.7)	
CEBPA, n (%)						
Single mutation	1 (2.2)	2 (4.4)	1.000	0	5 (13.9)	0.054
Double mutation	0	0		1 (2.8)	2 (5.6)	1.000
Wild type	44 (97.8)	43 (95.6)	1.000	35 (97.2)	29 (80.6)	0.055
DNMT3A, n (%)			1000			0.786
Mutation	13 (28.9)	12 (26.7)		10 (27.8)	8 (22.2)	
Wild type	32 (71.1)	33 (73.3)		26 (72.2)	28 (7.8)	
IDH1/IDH2, n (%)			0.167			0.415
Mutation	5 (11.1)	11 (24.4)		11 (30.6)	7 (19.4)	
Wild type	40 (88.9)	34 (75.6)		25 (69.4)	29 (80.6)	
RUNX1, n (%)			0.714			0.260
Mutation	5 (11.1)	3 (6.7)		6 (16.7)	2 (5.6)	
Wild type	40 (88.9)	42 (93.3)		30 (83.3)	34 (94.4)	
MLL–PTD, n (%)			1.000			0.614
Presence	2 (4.4)	3 (6.7)		3 (8.3)	1 (2.8)	
Absence	43 (95.6)	42 (93.7)		33 (91.7)	35 (97.2)	
NRAS/KRAS, n (%)			1.000			1.000
Mutation	6 (13.3)	7 (15.6)		4 (11.1)	3 (8.3)	
Wild type	39 (86.7)	38 (84.4)		32 (88.9)	33 (91.7)	
TET2, n (%)			0.118			0.614
Mutation	9 (20.0)	3 (6.7)		1 (2.8)	3 (8.3)	
Wild type	36 (80.0)	42 (93.7)		35 (97.2)	33 (91.7)	
TP53, n (%)			0.000			0.115
Mutation	11 (24.4)	0		4 (11.1)	0	
Wild type	34 (75.6)	45 (100.0)		32 (88.9)	36 (100.0)	
Relapse, n (%)			0.001			0.474
Yes	42 (93.3)	28 (62.2)		23 (63.9)	19 (47.2)	
No	3 (6.7)	17 (37.8)		13 (36.1)	17 (52.8)	

Mann–Whitney test was used for continuous variables. Chi square tests were used for categorical variables

*WBC* white blood cell, *BM* bone marrow, *PB* peripheral blood, *FAB* French–American–British classification

### Prognostic value of miR-363 expression in patients treated with chemotherapy or allo-HSCT

**Fig. 1:**
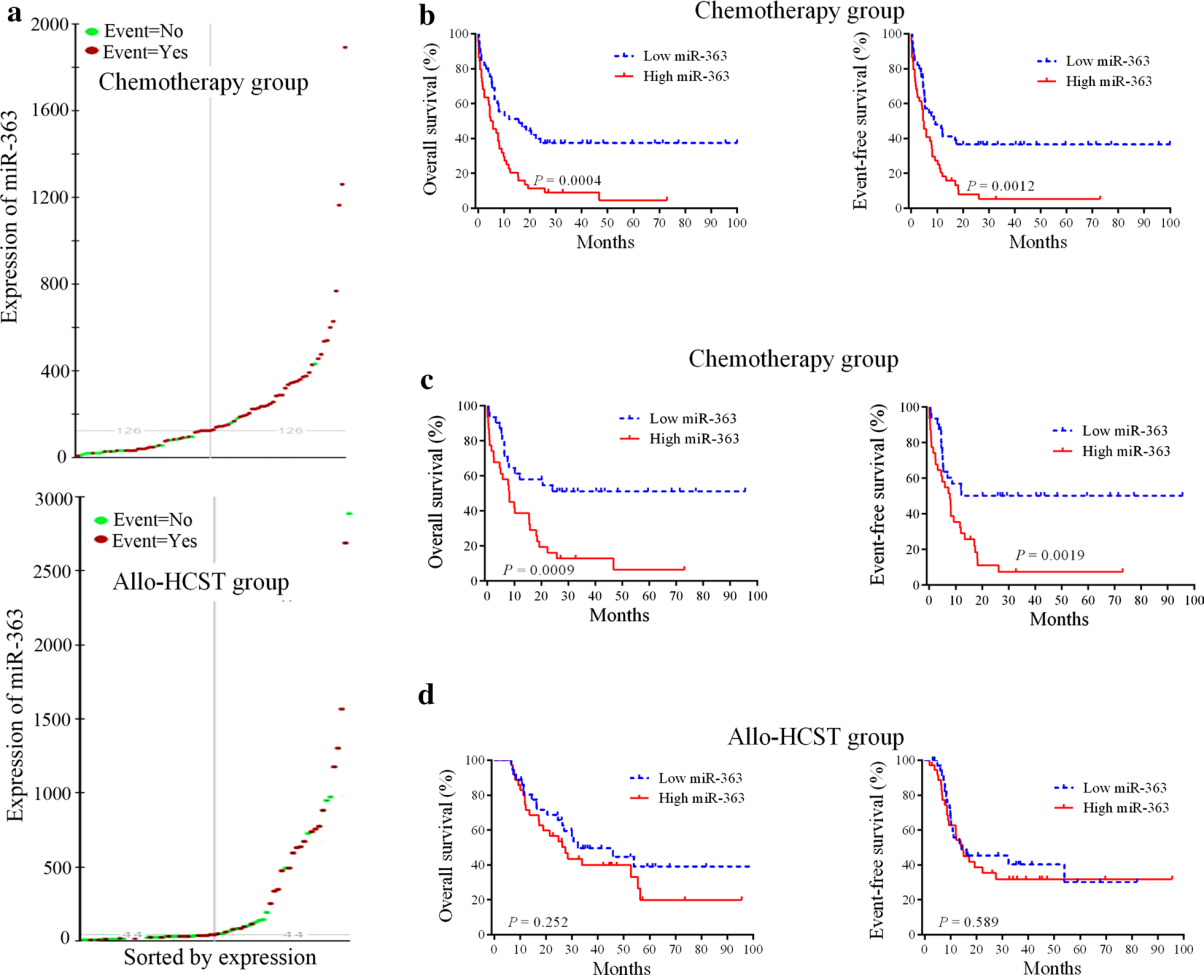
Kaplan–Meier survival curves based on miR-363 expression. **a** The expression level distribution of miR-363. **b** Cases highly expressing miR-363 showed markedly shorter OS and EFS in the chemotherapy group (n = 90). **c** Patients with high miR-363 expression had poor OS and EFS in the chemotherapy group. **d** Effect of miR-363 levels on OS and EFS in cases administered allo-HSCT (n = 72)

We performed genome-wide screening of miRNAs in AML cases in order to acquire prognostic marker to improve the classification of AML. MiR-363 was identified as a new prognostic marker for chemotherapy in AML patients. In order to evaluate survival of patients, we employed the Kaplan–Meier method and log-rank test. The expression level distribution of miR-363 was shown in Fig. 1a. In the chemotherapy group, cases highly expressing miR-363 showed reduced OS (HR = 2.28, *P* = 0.0004) and EFS (HR = 2.14, *P* = 0.0012) in comparison with low expressers (Fig. 1b). We further performed a survival analysis in the good/intermediate group, patients with high miR-363 expression had significantly shorter OS (*P* = 0.0009) and EFS (*P* = 0.0019) compared with patients with low miR-363 expression (Fig. 1c). However, miR-363 expression level was not associated with outcome in AML patients treated with allo-HCST (Fig. 1d). These data suggested that high expression of miR-363 was a poor prognostic factor in AML patients treated with chemotherapy.

### MiR-363 is associated with clinical outcome in AML

Univariate and multivariate cox analyses were performed to assess whether miR-363 level is an independent predictor of survival in AML. Univariate analysis (Table 2) showed that high miR-363 had an adverse prognostic value for predicting OS (HR = 2.389, *P* < 0.001) and EFS (HR = 2.224, *P* = 0.001) in cases administered chemotherapy. In multivariable analysis, miR-363 and multiple demonstrated prognostic factors were assessed (Table 2). High miR-363 remained an independent predictor of shorter OS (HR = 2.349, 95% CI 1.305–4.229, *P* = 0.012) and EFS (HR = 2.082, 95% CI 1.172–3.699, *P* = 0.001).

**Table 2:** Univariate and multivariate analyses in patients treated with chemotherapy

Variables	EFS		OS	
	HR (95% CI)	*P*-value	HR (95% CI)	*P*-value
Univariate analyses				
MiR-363 (high vs. low)	2.224 (1.369–3.612)	0.001	2.389 (1.468–3.887)	0.000
WBC (≥ 20 vs. < 20 × 10^9^/L)	1.015 (0.633–1.627)	0.952	0.980 (0.611–1.571)	0.932
FLT3–ITD (positive vs. negative)	1.095 (0.587–2.040)	0.776	1.049 (0.563–1.956)	0.880
NPM1 (mutated vs. wild)	1.050 (0.633–1.741)	0.850	0.965 (0.582–1.599)	0.890
DNMT3A (mutated vs. wild)	1.301 (0.774–2.185)	0.320	1.299 (0.775–2.179)	0.321
RUNX1 (mutated vs. wild)	1.502 (0.717–3.147)	0.281	1.591 (0.759–3.335)	0.219
TP53 (mutated vs. wild)	3.011 (1.539–5.892)	0.001	2.898 (1.487–5.649)	0.002
TET2 (mutated vs. wild)	0.778 (0.372–1.625)	0.504	0.686 (0.328–1.434)	0.316
MLL–PTD (mutated vs. wild)	0.891 (0.324–2.445)	0.822	0.945 (0.344–2.596)	0.913
IDH1/IDH2 (mutated vs. wild)	0.973 (0.271–1.273)	0.926	0.988 (0.550–1.777)	0.969
NRAS/KRAS (mutated vs. wild)	1.214 (0.637–2.314)	0.556	1.228 (0.644–2.340)	0.532
Multivariate analyses				
MiR-363 (high vs. low)	2.362 (1.346–4.145)	0.003	2.683 (1.507–4.779)	0.001
WBC (≥ 20 vs. < 20 × 10^9^/L)	1.806 (1.036–3.151)	0.037	1.861 (1.056–3.280)	0.032
RUNX1 (mutated vs. wild)	1.706 (0.797–3.654)	0.169	1.819 (0.850–3.892)	0.123
TP53 (mutated vs. wild)	2.786 (1.312–5.915)	0.008	2.566 (1.221–5.395)	0.013

*EFS* event-free survival, *OS* overall survival, *WBC* white blood cell

In patients receiving allo-HSCT, univariate analysis showed that adverse OS in patients with TP53-mutant. However, miR-363 expression status was not associated with OS and EFS in the allo-HSCT group (Table 3). Multivariable analysis revealed that TP53 and FLT3–ITD mutations independently predict adverse OS (*P* = 0.002 and *P* = 0.049, respectively). The miR-363 expression status did not persist as OS and EFS predictors in multivariable analysis.

**Table 3:** Univariate and multivariate analyses in patients treated with allo-HSCT

Variables	EFS		OS	
	HR (95% CI)	*P*-value	HR (95% CI)	*P*-value
Univariate analyses				
MiR-363 (high vs. low)	1.182 (0.643–2.175)	0.590	1.424 (0.775–2.619)	0.255
WBC (≥ 20 vs. < 20 × 10^9^/L)	1.089 (0.594–1.999)	0.782	0.826 (0.450–1.516)	0.537
FLT3–ITD (positive vs. negative)	1.876 (0.914–3.851)	0.086	1.973 (0.953–4.084)	0.067
NPM1 (mutated vs. wild)	1.007 (0.515–1.970)	0.983	1.023 (0.523–1.998)	0.948
DNMT3A (mutated vs. wild)	1.285 (0.655–2.520)	0.466	1.387 (0.704–2.731)	0.344
RUNX1 (mutated vs. wild)	1.145 (0.449–2.290)	0.777	1.579 (0.613–4.067)	0.344
TP53 (mutated vs. wild)	2.034 (0.718–5.760)	0.181	4.334 (1.453–12.925)	0.009
TET2 (mutated vs. wild)	0.526 (0.127–2.186)	0.377	0.670 (0.162–2.776)	0.581
MLL–PTD (mutated vs. wild)	5.775 (1.664–20.042)	0.006	2.728 (0.832–8.944)	0.098
IDH1/IDH2 (mutated vs. wild)	0.587 (0.271–1.273)	0.177	0.633 (0.293–1.368)	0.245
NRAS/KRAS (mutated vs. wild)	0.796 (0.245–2.586)	0.705	0.488 (0.150–1.587)	0.233
Multivariate analyses				
MLL–PTD (mutated vs. wild)	5.180 (1.449–18.511)	0.011	3.136 (0.943–10.429)	0.062
FLT3–ITD (positive vs. negative)	1.837 (0.868–3.888)	0.112	2.301 (1.090–4.860)	0.029
TP53 (mutated vs. wild)	2.493 (0.860–7.226)	0.092	5.848 (1.885–18.142)	0.002

*EFS* event-free survival, *OS* overall survival, *WBC* white blood cell

### Allo-HSCT overcomes the adverse prognostic role of miR-363 expression

**Fig. 2:**
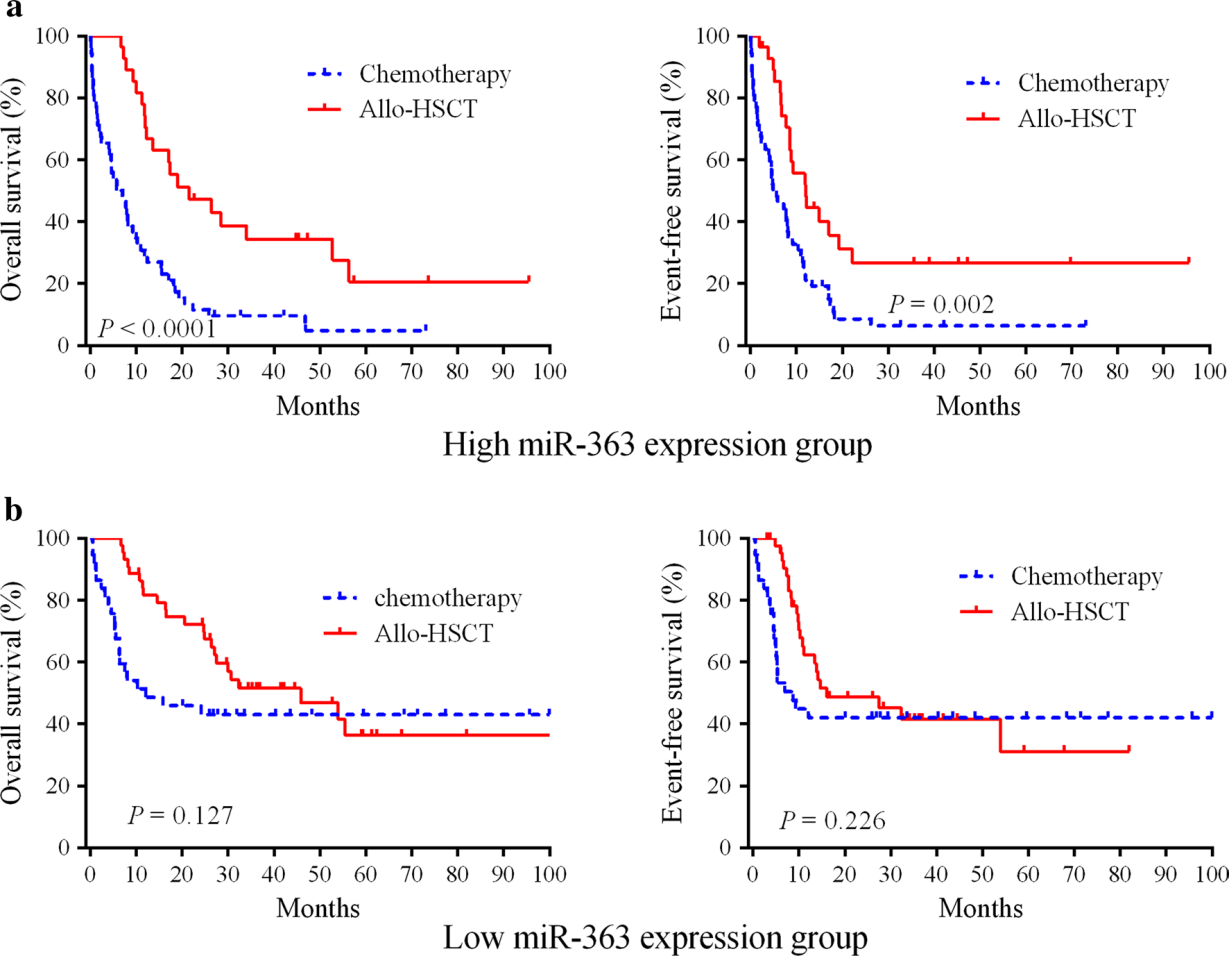
Allo-HSCT overcomes the adverse prognostic influence of high miR-363 expression in AML. **a** The 162 cases were divided into two groups according to median miR-363 levels. Kaplan–Meier survival curves for cases administered chemotherapy (n = 53) and allo-HSCT (n = 28), respectively, in the high miR-363 group. **b** Kaplan–Meier survival curves for cases administered chemotherapy (n = 37) and allo-HSCT (n = 44), respectively, in the low miR-363 group

Next, we investigated whether allo-HSCT could overcome the adverse outcomes of miR-363 expression. The 162 patients were divided into 2 groups according to the median level of miR-363. In the high miR-363 group, cases administered allo-HSCT showed markedly improved OS (HR = 0.361, 95% CI 0.225–0.588, *P* < 0.0001) and EFS (HR = 0.447, 95% CI 0.287–0.751, *P* = 0.002) in comparison with cases administered chemotherapy (Fig. 2a). In patients with lower expression of miR-363, no marked differences in OS (*P* = 0.127) and EFS (*P* = 0.226) were found between the chemotherapy and allo-HSCT groups (Fig. 2b). These results suggested that miR-363 may be considered as a prognostic marker for the detection of patients requiring optimal therapeutic schedules.

### Biological insight of miR-363 expression in AML

**Fig. 3:**
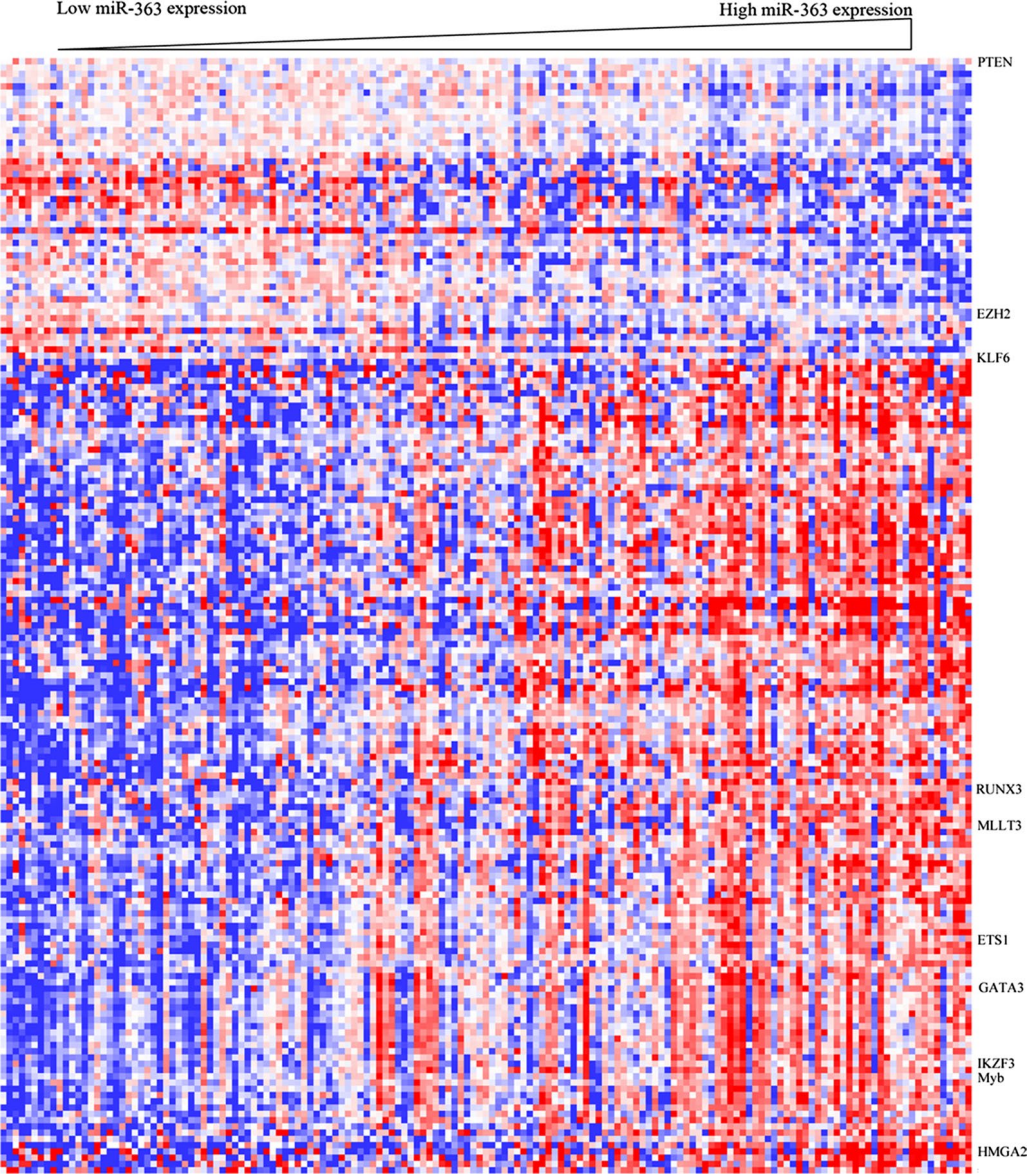
Heat map of the gene expression signature related to miR-363 expression in AML. Cases (columns) were ordered from left to right by increasing miR-363 levels. Genes (rows) were ordered by hierarchical cluster analysis. Blue and red reflect expression levels below and above median values for respective genes, respectively; miR-363 associated genes are indicated

To further investigate the biological function of miR-363, gene expression signature associated with miRNA expression was determined in AML cases. We observed that the levels of 178 genes were strongly associated with miR-363 expression, including 130 and 48 with positive and negative correlations, respectively (Fig. 3). Differentially upregulated genes in patients with high miR-363 expression included leukemogenic transcription factors (Myb, RUNX3, GATA3, IKZF3, HMGA2 and ETS1) [10, 11]. Notably, MLLT3/AF9 was up-regulated in the high miR-363 group, as a frequent fusion partner of the MLL gene in translocations t(9;11)(p22;q23) related to AML [12]. Among downregulated genes, we found that miR-363 expression showed negative correlations with the levels of tumor suppressor genes (EZH2, KLF6 and PTEN). Interestingly, these three genes were predicted miR-363 targets according to in silico analysis. Gene Ontology showed that genes associated with cell migration, T cell activation, system development, cell differentiation, response to chemicals and immune response were significantly correlated with miR-363 expression (Table 4). Thus, the miR-363 associated gene-expression profiling signature supported clinical finding in AML obtained by miRNA analysis.

**Table 4:** Gene ontology terms of biological processes in the miR-363 associated expression profile

GO ID	GO terms	Percentage of members of the GO term present in the miR-363 profile	*P*-value
GO:0050789	Regulation of biological process	69.5	< 0.001
GO:0050794	Regulation of cellular process	64.4	< 0.001
GO:0051716	Cellular response to stimulus	44.1	0.009
GO:0007154	Cell communication	40.7	0.009
GO:0032502	Developmental process	39.0	0.002
GO:0007275	Multicellular organism development	37.3	< 0.001
GO:0048731	System development	33.9	< 0.001
GO:0048869	Cellular developmental process	29.4	0.003
GO:0030154	Cell differentiation	28.2	0.001
GO:0042221	Response to chemical	27.7	0.025
GO:0010033	Response to organic substance	20.3	0.026
GO:0070887	Cellular response to chemical stimulus	19.8	0.018
GO:0009605	Response to external stimulus	16.9	0.009
GO:0045595	Regulation of cell differentiation	15.3	0.001
GO:0006955	Immune response	13.6	0.012

*GO* gene ontology

## Discussion

As current molecular stratification schemes do not fully grasp the heterogeneity of prognosis in patients with AML, the identification of novel prognostic markers is urgent [13]. In heterogeneous cohorts of AML patients, the correlation of miRNAs as predictive molecular markers remains largely unknown. In this study, miR-363 was determined as an independent prognostic factor in AML cases undergoing chemotherapy. Meanwhile, the miR-363 expression provides a powerful tool for risk stratification of AML patients. More importantly, allo-HSCT can overcome miR-363 expression-associated adverse outcomes.

We showed that miR-363 expression levels constitute independent prognostic marker of AML in a heterogeneous cohort administered chemotherapy. High miR-363 levels could still predict adverse outcome after consideration of other molecular prognostic factors in multivariable analysis. Thus, miR-363 could increase the prognostic value of previously defined molecular factors in a highly heterogeneous cohort of AML cases. Strikingly, patients with high miR-363 expression levels showed markedly poor OS and EFS. These findings suggest that miR-363 independently influences treatment outcomes and may synergistically drive leukemogenesis. More importantly, miR-363 expression levels could be useful to the identification of patients with adverse outcome in AML patients administrated chemotherapy.

Conventional chemotherapy and allo-HCST constitute the standard post-remission treatment strategies for AML [14]. However, there is a lack of efficient prognostic markers for guiding rational treatment options. We found that high miR-363 expressers administered allo-HSCT showed markedly improved OS and EFS in comparison with cases administered chemotherapy. In cases lowly expressing miR-363, there was no advantage for those administered allo-HSCT in comparison with the chemotherapy group. These findings indicate that patients with low miR-363 expression may not benefit from allo-HSCT as first-line therapy. Therefore, the expression of miR-363 may contribute to identify patients in need of strategies to select the optimal treatment regimen between chemotherapy and allo-HCST. The AML patients with high miR-363 expression may be preferably recommended for early allo-HSCT.

The possible oncogenic function of miR-363 has been reported previously in T-cell acute lymphoblastic leukemia, multiple myeloma and solid tumors [15, 16]. MiR-363 promotes growth and chemo-resistance in gastric adenocarcinoma by downregulating FBW7 [17]. It was shown that miR-363 is a prognostic marker for hepatocellular carcinoma [18]. However, the function and prognostic role of miR-363 in AML remains unclear. To derive biological insights from AML cases characterized by high miR-363 expression, we identified genes associated with miR-363 expression in AML patients. Interestingly, miR-363 expression was positively correlated with the amounts of leukemogenic transcription factors, including Myb, RUNX3, GATA3, IKZF3, ETS1 and MLLT3. The Myb oncogene, a driver of leukemogenesis, is widely expressed in AML and important for continued proliferation and differentiation blocking activity in AML cells [19]. ETS1 is critical in cell proliferation and differentiation in AML [20]. MLLT3 represents a commonly encountered fusion partner of MLL in translocations t(9;11)(p22;q23), which are related to AML [21]. Notably, we found that the direct target genes (EZH2, KLF6 and PTEN) of miR-363 were downregulated in association with high miR-363 expression. It was shown that loss-of-function mutations of the tumor suppressor gene EZH2 are found in AML [22]. Meanwhile, PTEN plays an essential role in the prevention of leukemogenesis [23, 24]. Indeed, PTEN deletion in hematopoietic cells can induce a myeloproliferative disease within days and transplantable leukemias within weeks. These miR-363 associated genes may participate in the adverse response to chemotherapy in cases highly expressing miR-363. Therefore, the miR-363 related gene-expression profiling signature may support the clinical observation that AML is characterized by the expression of miRNA. However, the mechanisms concerning the regulation of miR-363 expression and subsequent influence of AML treatment outcome remain to be elucidated.

## Conclusions

In conclusion, miR-363 levels independently correlate with clinical outcome in a highly heterogeneous cohort of AML cases. MiR-363 expression could greatly contribute to the identification of patients with poor outcome in AML. Expression analysis of miR-363 may be useful to improve the risk stratification of AML patients. Furthermore, allo-HSCT may overcome the unfavorable consequences of high miR-363 expression in AML. Therefore, the expression analysis of miR-363 may help identify cases in need of strategies to select the optimal treatment regimen between chemotherapy and allo-HCST.

## Abbreviations

AML: acute myeloid leukemia
Allo-HSCT: allogeneic hematopoietic stem cell transplantation
TCGA: The Cancer Genome Atlas
OS: overall survival
EFS: event-free survival
